# Mapping protein networks in yeast mitochondria using proximity-dependent biotin identification coupled to proteomics

**DOI:** 10.1016/j.xpro.2020.100219

**Published:** 2020-12-15

**Authors:** Roger Salvatori, Wasim Aftab, Ignasi Forne, Axel Imhof, Martin Ott, Abeer Prakash Singh

**Affiliations:** 1 Department of Biochemistry and Biophysics, Stockholm University, Stockholm 10691, Sweden; 2 Department of Medical Biochemistry and Cell Biology, University of Gothenburg, Gothenburg 40530, Sweden; 3 BioMedical Center, Faculty of Medicine, Ludwig Maximilians University of Munich, Planegg-Martinsried 82152, Germany; 4 Graduate School for Quantitative Biosciences (QBM), Ludwig Maximilians University of Munich, Munich 81377, Germany

**Keywords:** Bioinformatics, Molecular Biology, Protein Biochemistry, Proteomics

## Abstract

Proximity-dependent biotin identification (BioID) permits biotinylation of proteins interacting directly, indirectly, or just localized in proximity of a protein of interest (bait). Here, we describe how BioID coupled to proteomics and network biology can be used to map protein proximities in yeast mitochondria, aiding in visualization of complex protein-protein interaction landscapes.

For complete information on the use and execution of this protocol, please refer to Singh et al., 2020.

## Highlights

• Generation of cells with a chromosomal C-terminal BirA∗ tagged protein for BioID
• Biotin labeling and denaturing protein purification for mass spectrometry
• Automated quantitative analyses of proteomic data using R programming
• Generation of detailed networks to illustrate protein-protein proximities

## Graphical Abstract

**Figure:**
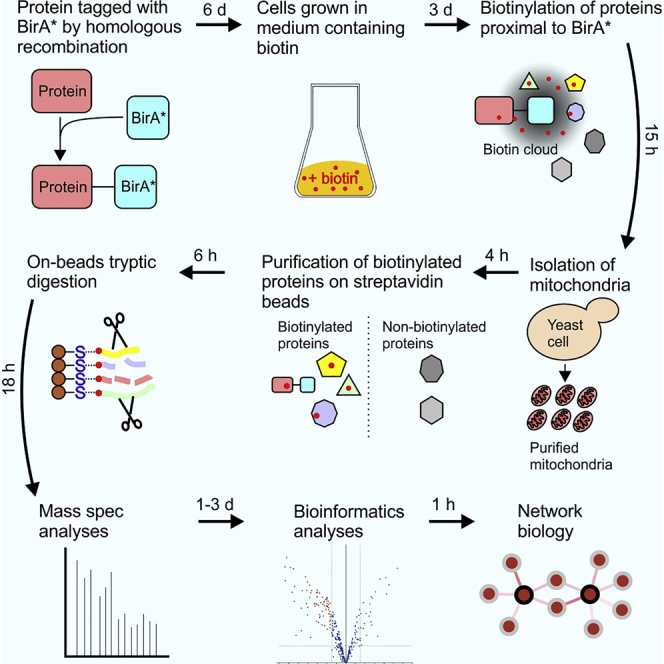

## Before you begin

Here we describe a proximity labeling technique optimized for studying proteins in yeast mitochondria. Importantly, BioID (*bio*tin *id*entification) permits the identification of transiently interacting proteins and also proteins located in vicinity of the bait, which may not be co-purified by conventional pull down experiments (Roux et al., 2012; Sears et al., 2019). Generally, BioID is based on the biotinylation performed by a mutated form of a biotin ligase of bacterial origin, BirA. The modified version of BirA used in BioID (BirA∗) carries a mutation (R118G), which consents promiscuous biotinylation of the proteins included within the labeling radius, calculated to be about 10 nm (Choi-Rhee et al., 2004; Kim et al., 2014). There are four essential aspects of this optimized protocol. First, the targeted protein is only present as a BirA∗-fused variant expressed from the authentic promotor. This excludes non-assembled (and hence non-physiological forms of the proteins) that can disturb the analyses; this is in turn important to detect proximity interactions of proteins assembled into functional complexes. Second, to increase sensitivity and specificity, mitochondria are prepared prior to isolation of the biotinylated proteins. Third, proteins are purified under stringent denaturing conditions (which again decreases the likelihood to purify intact complexes), resulting in increased specificity. Fourth, subsequent mass spectrometric and bioinformatics analyses are streamlined for facile visualization and coherent statistical treatment.

### Primer design and PCR for homologous recombination mediated C-terminal BirA∗ tagging

**Timing: 1 day**

**Figure 1:**
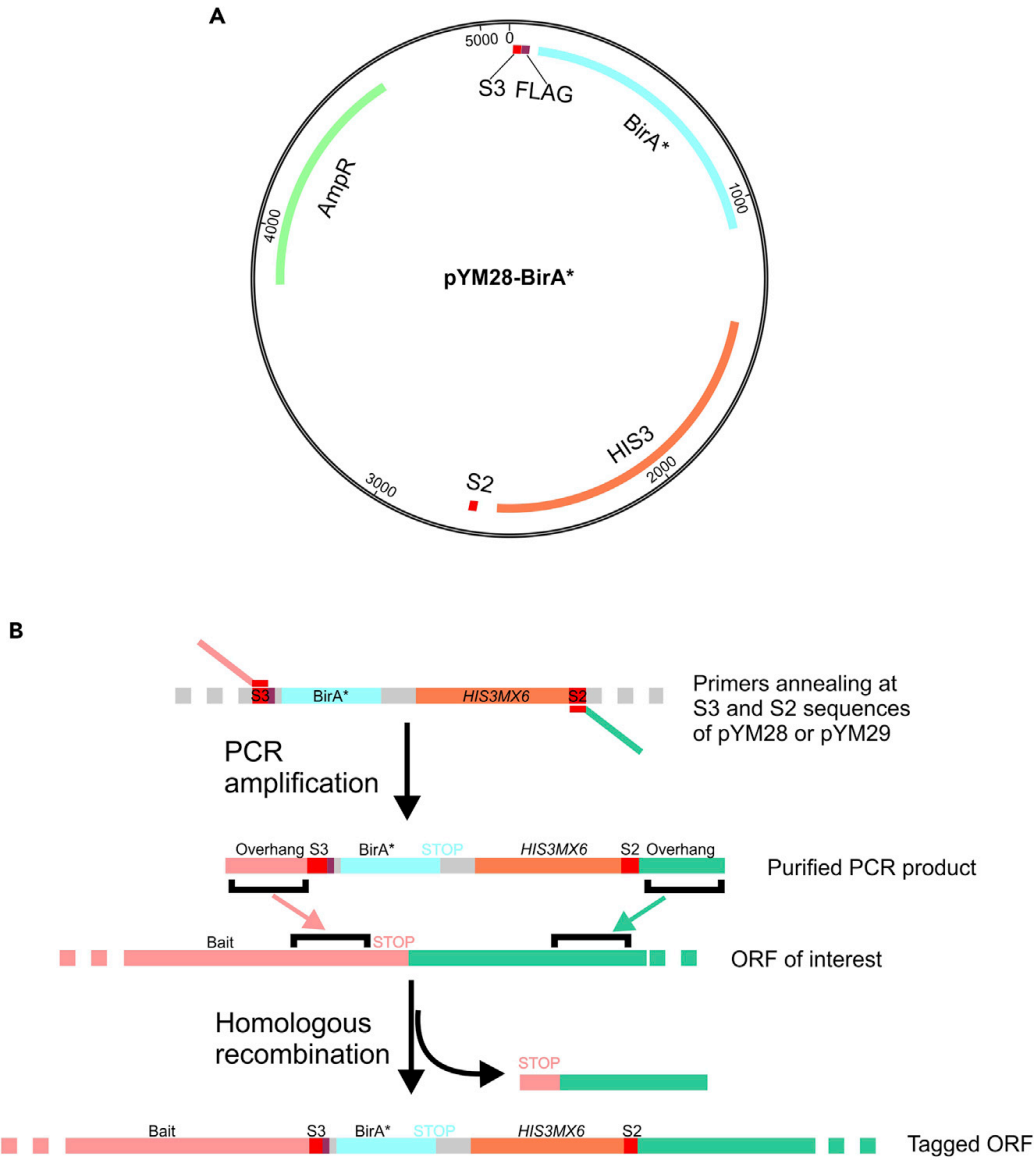
Construction of a plasmid for C-terminal BirA∗-tagging of a protein of interest (A) The sequence encoding BirA∗ tag was inserted into the plasmid pYM28 carrying an *HIS3* auxotrophic marker. (B) A segment of the plasmid including linker sequence, BirA∗ coding sequence, stop codon, and the auxotrophic marker is amplified by PCR. Then, this DNA segment is inserted by homologous recombination into the genomic DNA, thereby fusing BirA∗ to the C terminus of the protein of interest.

The coding sequence of BirA∗ was inserted into a pYM28 or a pYM29 plasmid backbone, permitting C-terminal tagging of the proteins of interest, following well-described procedures (Janke et al., 2004). Importantly, the tagged protein is expressed at authentic expression levels, avoiding undesired secondary effects caused by its overexpression. The two plasmids, which are available from Addgene (Addgene IDs: 160290 and 160291), contain cassettes for auxotrophic selection on media lacking histidine or tryptophan, respectively (Figure 1A). The BirA∗ is expressed fused to the native protein, separated by a short linker (CGTACGCTGCAGGTCGACLRTLQVDDYKDDDDKTRGGGGSGGGGS), which permits flexibility between the protein and the BirA∗-tag, ensuring full native activity of the bait protein and efficient biotinylation coverage (Osellame et al., 2016). Moreover, the linker contains a FLAG tag allowing for detection via western blotting or immunofluorescence. The PCR cassette that is used to transform yeast will finally contain a S3 sequence, the linker, the BirA∗ coding sequence followed by a stop codon and a terminator, the *HIS3* or *TRP1* gene with their respective promoter and terminator, and the S2 sequence (Figure 1B).

1. Primer design

Primers must contain at their 5′ ends homologous sequences to the gene of interest. More specifically, the forward primer must contain an overhang of about 40–51 nucleotides ending at the penultimate codon of the open reading frame and followed by S3 sequence (5′-CGTACGCTGCAGGTCGAC-3′). The reverse primer features the S2 sequence (5′-ATCGATGAATTCGAGCTCG-3′) at the 3′ end and, at 5′ end, an overhang downstream (at least 70 bp) of the stop codon of the protein of interest. The size of the overhang should be about 40–51 nucleotides. Hence, following homologous recombination, the native stop codon of the gene of interest is replaced by **5**′**-S3 sequence → linker → BirA∗ → STOP → terminator→ HIS/TRP selection marker → S2-3**′ sequence.

**CRITICAL:** Make sure that the region between the stop codon and the reverse primer binding site does not contain promotor/terminator sequences of adjacent genes. Typically, if the region is >200 bp further from the ORF of the adjacent gene, that would be considered appropriate for homologous recombination. For tagging of Mrpl4 we used Forward primer “Mrpl4_S3”: 5′-AAATACTTGAAACAGCTAATTCATGCAAGCTCCGTGGAGCAAGCAACTGCAcgtacgctgcaggtcgac-3′ and Reverse primer “Mrpl4_S2”: 5′-GAATACATACAGCGTATAGCTATATAAAGGTGTATGTATGTGTGTATGTATatcgatgaattcgagctcg-3′. The underlined bases correspond to the S3 and S2 sequences.

2. Set up the PCR reaction as follows.

**Table:** 

Component	Volume
5× Q5 reaction buffer	20 μL
10 mM dNTPs	2 μL
10 μM forward primer	5 μL
10 μM reverse primer	5 μL
Template plasmid	Equivalent to 50 ng
Q5 high-fidelity DNA polymerase	1 μL
Nuclease-free water	To 100 μL

3. Run the PCR on the following cycling conditions.

**Table:** 

PCR cycling conditions			
Steps	Temperature	Time	Cycles
Initial denaturation	98°C	30 s	1
Denaturation	98°C	10 s	30 cycles
Annealing	55°C–60°C	30 s	
Extension	72°C	2 min	
Final extension	72°C	2 min	1
Hold	12°C	Infinity

***Note:*** This protocol is for Q5 polymerase (NEB).

4. PCR product purification

Use any of the commercially available PCR clean-up kits for this step. The PCR product is dissolved in sterile deionized H_2_O and ready for transfection.

## Key resources table

**Table:** 

REAGENT or RESOURCE	SOURCE	IDENTIFIER
**Antibodies**		

Anti-BirA∗	Novus Biological	NBP2-59938

**Chemicals, peptides, and recombinant proteins**		

Lithium acetate	Sigma	L6883
PEG (poly ethylene glycol)	Sigma	P4338
SSDNA (deoxyribonucleic acid sodium salt from salmon testes)	Sigma	D1626
Tryptone/peptone	Roth	8952.5
Yeast extract	Serva	24540.03
Dextrose	Sigma	G8270
Galactose	Roth	4987.7
Glycerol	VWR	24387.292
Ponceau staining	Sigma	P3504
Skim milk powder	Sigma	70166
EDTA	Sigma	E5134
PMSF	Applichem	A0999,0025
Sorbitol	Roth	6213.2
Zymolyase 20T	Amsbio	120491-1
Biotin	Sigma	B4639
Streptavidin magnetic beads	Thermo Fisher Scientific	88817
16-tube SureBeads magnetic rack	Bio-Rad	1614916
Sequencing grade modified trypsin	Promega	V5111
Benzonase nuclease	Merck	E1014-5KU
3M Empore SDB-RPS extraction disks (2241)	Sigma	66886-U
LC-MS grade water	Merck	1.15333.1000
Acetonitrile (ACN)	Merck	1.00029.1000
Methanol (MeOH)	Roth	AE71.1
Isopropanol (ISO)	Roth	AE73.1
Ammonia solution (25% wt/vol; NH_4_OH)	Merck	5330030050
Acetic acid (AcOH)	Roth	3738.1
Trifluoroacetic acid (TFA)	Thermo Scientific	85183

**Critical commercial assays**		

ECL detection kit WesternBright Quantum	advansta	K-12042-D10
ECL detection kit WesternBright Sirius	advansta	K-12043-D10
AttractSPEDisks Tips SDB-RPS-200 μL-T1-96/pk	AFFINISEP	Tips-RPS-M.T1.200.96
16-gauge Kel-F hub needle	Hamilton	90516

**Deposited data**		

Raw data and partially processed files for Mrpl4 and Cbp3 BioIDs	This study	https://github.com/wasimaftab/BioID_Proteomics
Raw data of western blotting (Mendeley)	This study	https://doi.org/10.17632/pw4f8w65pk.1

**Experimental models: cell lines**		

*S. cerevisiae*: strain MOY646 *Mat α; ura3-1; trp1Δ 2; leu2-3,112; his3-11,15; ade2-1; can1-100 nuc1::URA3*	Kehrein et al., 2015	N/A
*S. cerevisiae*: strain MOY1258 *Mat α; ura3-1; trp1Δ 2; leu2-3,112; his3-11,15; ade2-1; can1-100 nuc1::URA3 Mrpl4BirA∗::HIS3*	Singh et al., 2020	N/A
*S. cerevisiae*: strain MOY1392 *Mat α; ura3-1; trp1Δ 2; leu2-3,112; his3-11,15; ade2-1; can1-100 nuc1::URA3 CBP3BirA∗::HIS3*	Salvatori et al., 2020	N/A
*S. cerevisiae*: strain MOY1242 *Mat α; ura3-1; trp1Δ 2; leu2-3,112; his3-11,15; ade2-1; can1-100 nuc1::URA3 KGD4BirA∗::HIS3*	Singh et al., 2020	N/A

**Oligonucleotides**		

Mrpl4_S3	Merck	N/A
Mrpl4_S2	Merck	N/A

**Recombinant DNA**		

pYM28-BirA∗	Salvatori et al., 2020	160290
pYM29-BirA∗	Salvatori et al., 2020	160291

**Software and algorithms**		

Cytoscape	Shannon et al., 2003	https://cytoscape.org
Statistical data analysis pipeline	This study	https://github.com/wasimaftab/BioID_Proteomics
Microsoft Excel	Microsoft Corporation	https://products.office.com/en/excel
MaxQuant (MQ)	Max Plank institute of Biochemistry	https://www.maxquant.org/
R	The R Project for Statistical Computing	https://www.r-project.org/
Rstudio	RStudio Team (2015). RStudio: Integrated Development for R. RStudio, Inc., Boston	http://www.rstudio.com/.

## Materials and equipment

### LC-MS setup

#### Nanospray column

Pulled fused-silica 15-cm-length capillary from New Objective (FS360-75-8-N-20-C15, 360 μm × 75 μm, 8±1-μm i.d. tip), packed in-house with C18 material (ReproSil-Pur, 120 Å, C18-AQ, 2.4 μm; Dr. Maisch, cat. no. r124aq). Commercial columns with similar features can also be used.

#### Column oven for nanospray columns

Sonation, cat. no. PRSO-V1 or PRSO-V2 (40°C–50°C).

#### LC system

Ultimate 3000 RSLCnano systems (Thermo Fisher Scientific) operated at 300 nL/min, with buffer A 0.1% (v/v) formic acid and buffer B 80% (v/v) ACN/0.1% (v/v) formic acid and the gradient (interval in minutes/% buffer B) 0/4, 5/4, 55/50, 56/90, 61/90, 62/4, 90/4. Other LC systems capable of operating at nl/min range can be used as well.

#### Mass spectrometer

Q Exactive HF (Thermo Fisher Scientific). Other mass spectrometer capable of Data-Dependent Acquisition (DDA) with sufficiently high resolution and scan speed, like LTQ-Orbitrap Elite and Velos series, Orbitrap Tribid instruments, or TOF instruments can be used as well. Operate the Q Exactive HF in DDA positive mode with a survey full scan MS spectra (from m/z 375–1,600) acquired with resolution R = 60,000 at m/z 400 (AGC target of 3 × 10^6^, maximum ion time 60 ms). The 10 most intense peptide ions with charge states between 2 and 5 are sequentially isolated to a target value of 1 × 10^5^, R = 60,000 at m/z 400, maximum ion time 60 ms, isolation window 2.0 m/z and fragmented at 27% normalized collision energy. Typical mass spectrometric conditions are: spray voltage, 1.5 kV; no sheath and auxiliary gas flow; heated capillary temperature, 300°C; ion selection threshold, 33.000 counts, S-lens RF level 50.

#### Stock solution preparation

**Table:** YP media

Reagent	Final concentration (g/L)	Amount
Bacto tryptone	20 g/L	20 g
Yeast extract	10 g/L	10 g
ddH_2_O	N/A	Up to 1 L

***Note:*** autoclave the same day after preparation. YP can be stored at room temperature for long term.

**Table:** HR Buffer

Reagent	Final concentration	Amount
Polyethylene glycol 50%	33% v/w	240 μL
Lithium acetate 1 M	100 mM	36 μL
Salmon sperm (SS) DNA 10 mg/mL	138.88 ng/μL	5 μL
DNA cassette	2.7 ng/μL	1 μg
ddH_2_O	N/A	Up to 360 μL
**Total**	**N/A**	**360 μL**

***Note:*** autoclave the polyethylene glycol 50% solution, and sterile filter the Lithium acetate 1 M solution before use. All reagents can be stored at room temperature except for SS DNA, which is stored at −20°C.

**Table:** 2× SDS sample buffer

Reagent	Final concentration	Amount
Tris-HCl 1 M, pH 6.8	100 mM	5 mL
SDS	4%	2 g
Glycerol	20%	10 mL
Bromophenol blue	0.2%	10 mg
DTT 4 M	100 mM	1.25 mL
ddH_2_O	N/A	Up to 50 mL
**Total**	**N/A**	**50 mL**

***Note:*** Can be stored at −20°C for long time.

**Table:** TBS

Reagent	Final concentration	Amount
Tris-HCl pH 8.0	500 mM	60.5 g
Sodium chloride	1.5 M	87.6 g
Water	N/A	Up to 1 L
**Total**	**N/A**	**1 L**

***Note:*** Can be stored at room temperature for up to 1 year.

**Table:** MP1 buffer (2 mL/ g of wet cells)

Reagent	Final concentration (mM or μM)	Amount
Tris (unadjusted buffer) 1 M	100 mM	10 mL
1,4-dithiothreitol 4 M	10 mM	0.25 mL
ddH_2_O	N/A	89.75 mL
**Total**	**N/A**	**100 mL**

***Note:*** Used when made fresh.

**Table:** MP2 buffer (6.7 mL/g of wet cells)

Reagent	Final concentration (mM or μM)	Amount
Sorbitol 2.4 M	1.2 M	125 mL
Zymolyase	3 mg/g of wet weight of cells	120 mg
KPi 1 M, pH 7.4	20 mM	5 mL
ddH_2_O	N/A	120 mL
**Total**	**N/A**	**250 mL**

***Note:*** Used when made fresh.

**Table:** Homogenization buffer (13.4 mL/g of wet cells)

Reagent	Final concentration (mM or μM)	Amount
Tris-HCl 1 M, pH 7.4	10 mM	5 mL
PMSF 200 mM	1 mM	2.5 mL
EDTA 0.5 M, pH 8	1 mM	1 mL
Sorbitol 2.4 M	0.6 M	125 mL
ddH_2_O	N/A	366.5 mL
**Total**	**N/A**	**500 mL**

***Note:*** Used when made fresh.

**Table:** SH buffer

Reagent	Final concentration (mM or μM)	Amount
HEPES-KOH 1 M, pH 7.4	20 mM	1 mL
Sorbitol 2.4 M	600 mM	12.5 mL
ddH_2_O	N/A	36.5 mL
**Total**	**N/A**	**50 mL**

***Note:*** Used when made fresh.

**Table:** RIPA buffer

Reagent	Final concentration (mM or μM)	Amount
Tris-HCl, pH 7.5	50 mM	6.057 g
Sodium chloride	150 mM	8.766 g
Tergitol (NP-40)	1% w/v	10 g
EDTA	1 mM	0.292 g
EGTA	1 mM	0.380 g
SDS	0.1% w/v	1 g
Roche Protease cocktail inhibitor	1 tablet every 50 mL of buffer	N/A
Sodium deoxycholate	0.5% w/v	5 g
ddH_2_O	N/A	Up to 1,000 mL
**Total**	**N/A**	**1,000 mL**

***Note:*** Can be stored at 4°C for up to 4 months. Add Protease cocktail inhibitor fresh before use.

**Table:** TAP buffer

Reagent	Final concentration (mM or μM)	Amount
HEPES-KOH pH 8.0	50 mM	2.98 g
KCl	100 mM	1.86 g
Glycerol	10% v/v	25 mL
EDTA	2 mM	0.146 g
ddH_2_O	N/A	Up to 250 mL
**Total**	**N/A**	**250 mL**

***Note:*** Can be stored at 4°C for up to 4 months.

**Table:** ABC buffer

Reagent	Final concentration	Amount
Ammonium bicarbonate	50 mM	197 mg
Water	N/A	Up to 50 mL
**Total**	**N/A**	**50 mL**

***Note:*** Make fresh before use. Can be used up till 24 h.

**Table:** StageTip conditioning buffer 2

Reagent	Final concentration (mM or μM)	Amount
Methanol	30% v/v	3 mL
Trifluoroacetic acid	0.2% v/v	20 μL
LC-MS grade H_2_O	N/A	6.98 mL
**Total**	**N/A**	**10 mL**

***Caution:*** TFA solutions are corrosive. Prepare the solutions in a fume hood and handle with gloves. Make fresh before use.

**Table:** StageTip conditioning buffer 3/Stagetip wash buffer 2

Reagent	Final concentration (mM or μM)	Amount
Trifluoroacetic acid	0.2% v/v	20 μL
LC-MS grade H_2_O	N/A	9.98 mL
**Total**	**N/A**	**10 mL**

***Caution:*** TFA solutions are corrosive. Prepare the solutions in a fume hood and handle with gloves. Make fresh before use.

**Table:** StageTip wash buffer 1

Reagent	Final concentration (mM or μM)	Amount
Trifluoroacetic acid	1% v/v	0.1 μL
Isopropanol	99% v/v	9.9 mL
**Total**	**N/A**	**10 mL**

***Caution:*** TFA solutions are corrosive. Prepare the solutions in a fume hood and handle with gloves. Make fresh before use.

**Table:** StageTip elution buffer

Reagent	Final concentration (mM or μM)	Amount
ACN	80% v/v	8 mL
NH_4_OH (25%, HPLC grade)	1.25% v/v	0.5 mL
LC-MS grade H_2_O	N/A	1.500 mL
**Total**	**N/A**	**10 mL**

***Caution:*** This buffer must be prepared fresh. Otherwise, the pH will begin to increase due to the high volatility of NH_4_OH, and its elution strength will decrease.

**Table:** MS loading buffer

Reagent	Final concentration (mM or μM)	Amount
ACN	2% v/v	0.2 mL
Trifluoroacetic acid	0.3% v/v	30 μL
LC-MS grade H_2_O	N/A	9.77 mL
**Total**	**N/A**	**10 mL**

***Caution:*** TFA solutions are corrosive. Prepare the solutions in a fume hood and handle with gloves. Make fresh before use.

## Step-by-step method details

### Tagging the protein of interest via homologous recombination

**Timing: 6 days**

This step is required to genomically tag BirA∗ to the C-terminal of the protein of interest, using transfection mediated homologous recombination. Tagging is confirmed by Western blotting using anti-BirA antibody.

1. Grow the yeast cell line of interest for 12–16 h in YP + 2% dextrose.
2. Dilute the culture to 0.2 OD in YP + 2% dextrose and incubate under shaking at 30°C until the culture reaches an OD of ∼1.0.
3. Harvest 1.5 mL of cells by spinning at 3,000 × *g* for 5 min at ∼21°C.
4. Remove supernatant.
5. Resuspend cells in 1 mL of sterile water and spin at 3,000 × *g* for 5 min at ∼21°C.
6. Remove supernatant.
7. Perform PCR-tagging via homologous recombination.
  a) Resuspend the cells in 100 mM lithium acetate and shake them for 10 min at 30°C.
  b) Pellet the cells at 3,000 × *g* for 5 min and resuspend them by vortexing in 360 μL of HR buffer that includes the DNA fragment for homologous recombination.
  c) Incubate the cells at 42°C for 45 min under constant shaking.
  d) Spin down the cells at 3,000 × *g* at ∼21°C for 5 min, remove supernatant and resuspend them in 1 mL of YP + 2% dextrose.
  e) Incubate the cells for 1 h at 30°C under constant shaking.
  f) Spin down the cells at 3,000 × *g* at ∼21°C for 5 min and resuspend them in 100 μL of water, and spread them on an appropriate selective agar plate.
8. After three days (∼72 h), single-cell colonies should emerge on the plate.

**Pause Point:** the plates can be stored at 4°C for up to 2 weeks.

9. pick single colonies and streak them on a fresh selection plate and let them grow at 30°C for 36–48 h.

**Pause Point:** the plates can be stored at 4°C for up to 2 weeks.

10. Inoculate the clones in YP + 2% dextrose and let them grow for 12–16 h at 30°C under constant shaking.
11. Perform protein extraction.
  a) Harvest 1 OD of cells and spin them down.
  b) Resuspend the cells in 0.2 mL of 0.1 M NaOH and incubate at ∼21°C for 5 min.
  c) Spin the cells down at 3,000 × *g* for 5 min at ∼21°C and remove supernatant.
  d) Resuspend the cells in 30 μL of 1× SDS sample buffer.
  e) Boil the sample at 95°C for 5 min.
12. Load the sample on an SDS-PAGE gel.
13. Run the gel according to the manufacturer instructions.
14. Transfer the protein on a nitrocellulose membrane.
15. Stain the membrane with Ponceau S to confirm transfer.
16. Block the membrane with 5% skim milk powder in TBS for 1 h at ∼21°C.
17. Decorate the membrane with the anti-BirA antibody (Diluted 1:500 in 5% skim milk powder in TBS) on a shaker for 12–16 h at 4°C.
18. Wash the membrane three times with TBS.
19. Expose the membrane to the anti-mouse secondary antibody conjugated to HRP (Diluted 1:3,000 in 5% skim milk powder in TBS) for 1 h at ∼21°C.
20. Wash the membrane three times with TBS.
21. Incubate the membrane with ECL mix and check for correct BirA∗ tagging of the protein of interest.

### Labeling cells with biotin and extracting crude mitochondria

**Timing: 3–4 days**

In this step, biotin labeling of BirA∗-tagged cells and subsequently extract crude mitochondria is performed.

***Note:*** Perform three biological replicates for each strain.

22. Grow the cell line containing the BirA∗-tagged version of the protein of interest in YP + 2% glycerol or galactose supplemented with 50 μM biotin.
  a) Start the culture in 20 mL in YP + 2% glycerol or galactose and grow 12–16 h at 30°C under shaking.
  b) Increase the culture volume to 200 mL by supplementing YP + 2% glycerol or galactose.
  c) Bring the volume to 2 liters, add biotin powder into the culture to reach a final concentration of 50 μM and let the culture grow for ∼15 h at 30°C under shaking.

**CRITICAL:** It is important to harvest the cells for mitochondrial extraction when they are in logarithmic growth phase. The duplication time might be influenced by the strain’s genetic background. In our hands, starting a 2 liters culture with an OD_600_∼ 0.025–0.05 typically results on an OD ∼1.5 after 15 h.

23. When the OD_600_ is ∼1.5, harvest the cells by centrifuging at 3,000 × *g* for 5 min at ∼21°C.
24. Remove supernatant.
25. Resuspend the cells with water.
26. Spin down the cells at 2,900 × *g* at ∼21°C for 5 min. Remove supernatant.
27. Weight the wet cell pellet, expected to be around 6 grams for a 2 liters culture harvested at OD_600_ ∼1.5.
28. Resuspend the cells in MP1 buffer and incubate at 30°C for 10 min under shaking.
29. Spin down the cells at 2,900 × *g* at 21°C for 5 min and wash them with 1.2 M sorbitol.
30. Spin down the cells at 2,900 × *g* at 21°C for 5 min and resuspend them in MP2 buffer for 1 h at 30°C under shaking.
31. Disrupt the cells with a tight-fitting glass Dounce homogenizer.
  a) Spin down the cells at 3,000 × *g* at 4°C for 5 min and resuspend them in half of the required homogenization buffer.
  b) Dounce 10 times.
  c) Spin down the homogenate for 5 min at 2,900 × *g* at 4°C and save the supernatant at 4°C.
  d) Resuspend the pellet with the second half of homogenization buffer.
  e) Dounce 10 times.
  f) Spin down the homogenate for 5 min at 2,900 × *g* at 4°C and add this supernatant to the one saved before.
32. Spin down the total supernatant at 2,900 × *g* for 5 min at 4°C.
33. Transfer the supernatant in a fresh bottle.
34. Spin down the supernatant at 17,500 × *g* for 15 min at 4°C and discard the supernatant.
35. The pellet contains crude mitochondrial extract. Resuspend them in SH buffer (∼10 mg/mL) and fast freeze in liquid nitrogen followed by storage at −80°C.

**Pause Point:** mitochondria can be stored at −80°C for up to 1 year.

### Purification of biotinylated proteins

**Timing: 2 days**

In this step, total biotinylated proteins from the mitochondrial extract are purified using streptavidin magnetic beads and digested on-bead by trypsin. The peptides are then prepared for subsequent analysis by mass spectrometry.

36. Thaw mitochondria (3 mg of total protein per biological replicate) on ice or at 4°C.
37. Spin down the mitochondria for 10 min at 10,000 × *g* at 4°C.
38. Remove the supernatant and solubilize the mitochondria in 300 μL of 1% SDS, and incubate for 5 min at 50°C. Please see critical point below.

**CRITICAL:** This step is important as it will extract and denature all the proteins. This will reduce non-specific binding of proteins to streptavidin magnetic beads (step 43).

39. Dilute the mitochondrial lysate by adding 2,700 μL of RIPA buffer supplemented with 500 U of Benzonase and incubate for 30 min on ice (4°C).
40. Clarify the mitochondrial lysate by spinning at 16,000 × *g* for 5 min at 4°C.
41. Transfer the supernatant into a fresh 15 mL tube.
42. In the meanwhile, equilibrate streptavidin magnetic beads equivalent to 60 μL of beads slurry (to reach 50 μg protein/μL beads slurry) with 1 mL of RIPA buffer.
43. Spin down the beads, remove the supernatant, and transfer the equilibrated beads into the 15 mL tube with the mitochondrial lysate.
44. Incubate on a rotary shaker for 3 h at 4°C.
45. After the incubation, remove the supernatant (not bound fraction) by either centrifugation at 3,000 × *g* for 5 min at ∼21°C or by using a magnet.

**CRITICAL:** Make sure all the beads have been spun to the bottom of the tube as a pellet. Presence of beads in the supernatant will give a brown tinge to the solution.

46. Add 1 mL of RIPA buffer to wash the beads.
47. Transfer the beads in a fresh 1.5 mL tube and load onto a magnetic rack.

***Note:*** The magnet will attract the beads to one side of the tube.

48. Remove the RIPA buffer by carefully pipetting.
49. Wash again with 1 mL of RIPA buffer.
50. Wash the beads twice with 1 mL of TAP lysis buffer.
51. Wash the beads three times with 1 mL of freshly made ABC buffer.
52. Resuspend the beads with 200 μL of ABC buffer.
53. Add 1 μg of sequencing grade modified trypsin.
54. Incubate the tubes for 12–16 h at 37°C under constant shaking.
55. Add an additional 1 μg of sequence grade modified trypsin to the bead mixture, and incubate for 3 h at 37°C under constant shaking.
56. Harvest the elution (clear supernatant without the beads).
57. Resuspend the beads twice in 150 μL of ABC buffer, and harvest the supernatant.
58. Merge the three supernatants together (total volume= 500 μL).
59. Ensure the high purity of the elution by keeping the tubes on the magnetic rack for 5 min, and transferring the elutions in a new tube.

**Pause Point:** The elutions can be stored at −80°C for weeks before proceeding to the next step.

60. Lyophilize the elutions by solvent removal in a SpeedVac vacuum concentrator.
61. Resuspend the digested proteins in 30 μL of 0.1% TFA and incubate for 10 min at 4°C.
62. Check the pH. It should be between 2 and 3, otherwise adjust with 1% TFA.
63. Prepare StageTips with 2 layers of SDB-RPS per sample by piercing the SDB-RPS extraction disk with a 16-gauge Kel-F Hub Needle (90516 from Hamilton) and pushing the plugs down with a 1 mm OD metal wire into a 200 μL pipette tip, as described previously (Rappsilber et al., 2007). Commercial alternatives are also available (Tips-RPS-M.T1.200 from Affinisep).
64. Pre-equilibrate the StageTips sequentially with 50 μL ACN, 50 μL of Conditioning Buffer 2 and 50 μL of Conditioning Buffer 3 by centrifugation for 5–8 min at 1,500 × *g* at ∼21°C, or until no liquid remains in the StageTip.
65. Load each sample into a StageTip.
66. Wash the StageTips sequentially with Wash Buffer 1 and Wash Buffer 2 by centrifugation for 5–8 min at 1,500 × *g* at ∼21°C, or until no liquid remains in the StageTip.
67. Elute the peptides with 60 μL of elution buffer and collect the peptides into a clean low protein binding tube by centrifugation for 5 min at 500 × *g* at ∼21°C, or until no liquid remains in the StageTip.
68. Lyophilize the elutions by solvent removal in a vacuum concentrator for 30 min at 45°C, or until ∼2 μL remains.
69. Add 15 μL of MS loading buffer (2% ACN, 0.3% TFA) and resuspend by sonication.

**Pause Point:** The samples can be stored at −80°C for weeks before proceeding to the next step.

### Mass spectrometry and MaxQuant analysis

**Timing: 1–3 days**

In this step, the samples are processed by mass spectrometry and the raw data is analyzed by MaxQuant.

70. Place the samples in the LC autosampler cooled to 4–8°C and analyze 5 μL of each sample using the LC-MS setup as described in Material and Equipment.
71. Analyze the raw BioID data using MaxQuant (Cox and Mann, 2008; Tyanova et al., 2016) and perform the downstream bioinformatics analysis as described below.
72. Specify the parameters used for searching label-free BioID data using MaxQuant. Typical parameters for MQ 1.6.15.0 are described in the mqpar.xml file (https://github.com/wasimaftab/BioID_Proteomics/Example/MQ_param). See (Tyanova et al., 2016) for more details on MaxQuant settings.

***Note:*** The raw files of the proteomic results that we present in this manuscript was processed using MQ v1.5.2.8. Therefore, users attempting to reproduce the results with later versions of MQ might get slightly different results.

### Statistical quantification and generation of volcano plots

**Timing: 30–60 min**

In this step, two-group statistical quantification analysis is done on the Maxquant output data using a code developed using R programming. The code also generates volcano plots for easy visualization. To implement this analysis in your workflow, refer to the Quantification and statistical analysis section for a step-by-step description.

### Generation of protein-proximity networks

**Timing: 30–60 min**

In this step, fold-change enrichments from multiple BioID analyses are combined to create a bait-prey-edge table that can be visually analyzed as a network in Cytoscape.

73. Generating bait-prey-edge table
  a) If you run multiple two-group comparisons, you will end up creating multiple "*Results_timestamp*" folders. Inside each "*Results_timestamp*" folder there will be one "*timestamp_final_data.tsv*" file.
  b) Create a folder in your preferred location and copy those "timestamp_final_data.tsv" into that folder.
  c) Run the "*automate_nw_tab_gen.R*" code in RStudio by clicking **Source** and select the folder where you copied the .tsv files (see *Example/Limma_output/Final_tsvs* folder).
  d) The code will first ask users to specify a log fold-change cutoff. Then, for every .tsv file in that folder, it will print a list of iBAQ/LFQ column names and ask you to enter a bait name from that list. Here assumption is that iBAQ/LFQ columns will contain bait names.
  e) Using that information, the code will create a virtual list of bait-prey interactions that have fold change ≥ cutoff (e.g., 1.5) and p < 0.05.
  f) After that, the code will print a message if that file is processed successfully.
  g) Finally, when all .tsv files are processed successfully, code will concatenate those virtual lists into a single one, print top 10 rows that list and save all the proximity interactions as "Links.xlsx" in the same directory where you copied .tsv files earlier (see Example/Limma_output/Final_tsvs/Links.xlsx).
  h) To visualize a bait-prey interaction network, open Cytoscape and click File → Import → Network from File… and load "Links.xlsx."
  i) For “Bait” select Meaning: “Source Node” and Data type: “String.” For “Prey” select Meaning: “Target Node” and Data type: “String.” For “Fold change” select Meaning: “Edge attribute” and Data type: “Floating point.” Click “OK.”
  j) Click Edit → Remove Self-Loops… to remove self-interactions involving baits.
  k) The network displayed represents Bait-prey undirected network which can be customized depending on the user preference.

***Note:*** Tutorials for navigating through Cytoscape applications are available on GitHub by following this link: https://github.com/cytoscape/cytoscape-tutorials/wiki .This section of the protocol was tested on Windows 10 (64 bit) using Cytoscape 3.7.2.

***Optional:*** Bait-prey edge table can also be generated manually in Microsoft Excel. The above code automates the process and eliminates the risk of human error.

## Expected outcomes

Mitochondria contain proteins of dual genetic sources, and a small genome present in the mitochondrial matrix that is responsible for the production of a few key subunits of the oxidative phosphorylation system. Hence, assembly of respiratory chain complexes I, III, and IV requires expression of nuclear and mitochondrial encoded genes. To avoid accumulation of non-assembled subunits, the activities of both genetic systems need to be coordinated, which occurs by feedback loops that regulate mitochondrial translation. This translational control in mitochondria depends on the action of nuclear encoded factors called translational activators (Ott et al., 2016). The best studied translation feedback loops adjust translation of *COB* and *COX1*, two mRNAs coding for the cytochrome *b* subunit of complex III and subunit Cox1 of complex IV, respectively. The conserved Cbp3-Cbp6 complex interacts with newly synthesized cytochrome *b* to support its assembly and maturation (Gruschke et al., 2011; Gruschke et al., 2012). At the same time, the occupancy of Cbp3-Cbp6 with cytochrome *b* sets the levels of cytochrome *b* production. In cases where assembly proceeds, Cbp3-Cbp6 will dissociate from cytochrome *b* to stimulate a new round of *COB* mRNA translation (Gruschke et al., 2012; Hildenbeutel et al., 2014). To this end, free Cbp3-Cbp6 interacts with the tunnel exit of the mitochondrial ribosome to liberate *COB* mRNA and induce its translation (Salvatori et al., 2020). These results prompted the question whether mitochondrial ribosomes are specifically equipped with factors supporting the biogenesis of select mitochondrial encoded proteins.

**Figure 2:**
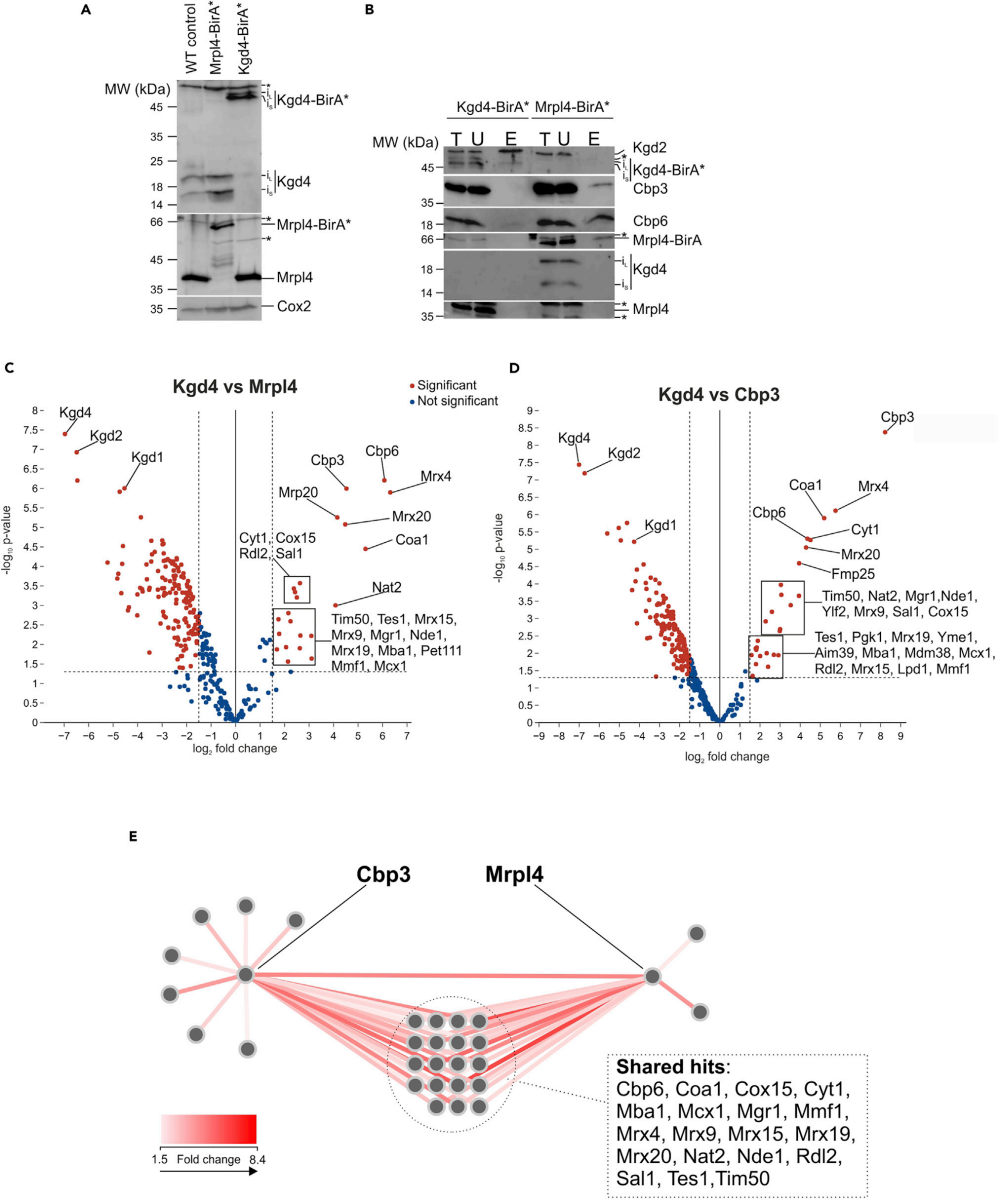
BioIDs of Mrpl4 and Cbp3 and generation of proximity interactome network (A) Western blots showing successful tagging of Mrpl4 and Kgd4 with BirA∗ by immunodecorating with anti-Mrpl4 and anti-Kgd4 antibody, respectively. Immunodecoration with anti-Cox2 shows that mitochondrial translation is not impacted in the BirA∗-tagged cell lines. (B) During purification of biotinylated proteins, instead of tryptic digestion, SDS-loading dye was added to the beads and boiled at 95°C for 20 min under shaking to elute the proteins. The lysate (T), unbound fraction (U), and elutions (E) were separated by SDS-PAGE followed by western blotting. (C and D) Volcano plots of Mrpl4 and Cbp3 individual BioIDs. (E) The enrichment data obtained from (C) and (D) were used to create a bait-prey network in Cytoscape; each protein is shown as a node, connect by red lines (edges) that are quantitatively color-mapped. ∗, cross-reactive bands; i_L_, long isoform of Kgd4; i_S_, short isoform of Kgd4.

In order to test this hypothesis, we used the above protocol to perform C-terminal BirA∗ tagging for Cbp3, Mrpl4, a mitoribosomal protein located at the rim of the polypeptide tunnel exit (Gruschke et al., 2010), and Kgd4, used as control (Figure 2A). Kgd4 is a soluble protein of the alpha ketoglutarate dehydrogenase complex present in the mitochondrial matrix (Heublein et al., 2014) and not directly involved in mitochondrial gene expression. In a pilot experiment, we purified biotinylated proteins from strains containing the BirA∗-tag on Mrpl4 and Kgd4 (Figure 2B). This led to the co-purification of Kgd2, a subunit of the mitochondrial alpha ketoglutarate dehydrogenase, in the case of Kgd4-BirA∗, and of Cbp3 in the case of Mrpl4-BirA∗. This latter observation confirms a previous finding obtained by chemical crosslinking showing that Cbp3 is found in close proximity to Mrpl4 (Gruschke et al., 2011).

Then, we applied BioID coupled to proteomics to determine Mrpl4 and Cbp3 proximity interactomes. As previously (Singh et al., 2020), we used Kgd4 as a contrasting bait to eliminate background, non-specific biotinylation and performed quantitative analysis. The results are presented in the two-side volcano plots, and the hits are considered significant if they have a fold-change enrichment of ≥1.5 and p <0.05 (Figures 2C and 2D). The BioID analyses of Kgd4 indeed revealed proximity to Kgd2 and Kgd1, two catalytic subunits of the alpha ketoglutarate dehydrogenase, further confirming that Kgd4 is part of the complex (Figures 2C and 2D, left side). Moreover, the proximity interactome of Mrpl4 contained Cbp3-Cbp6 together with ribosomal proteins and other respiratory chain assembly factors (Figure 2C, right side). Likewise, BioID analyses revealed the localization of various respiratory chain assembly factors in vicinity to Cbp3 (Figure 2D, right side). Finally, we created a protein-proximity network by merging the two BioIDomes into a bait-prey network (Figure 2E). This includes the significant hits from both Mrpl4 and Cbp3 BioIDs, revealing the common hits between the two baits. By combining multiple BioIDs, we can declutter complex biological processes into a comprehensive and elegant network that can aid in generating novel mechanistic insights, as previously demonstrated (Singh et al., 2020).

## Quantification and statistical analysis

The code performs two-group comparison in proteomics data available in GITHUB repository: https://github.com/wasimaftab/BioID_Proteomics.

**Figure 3:**
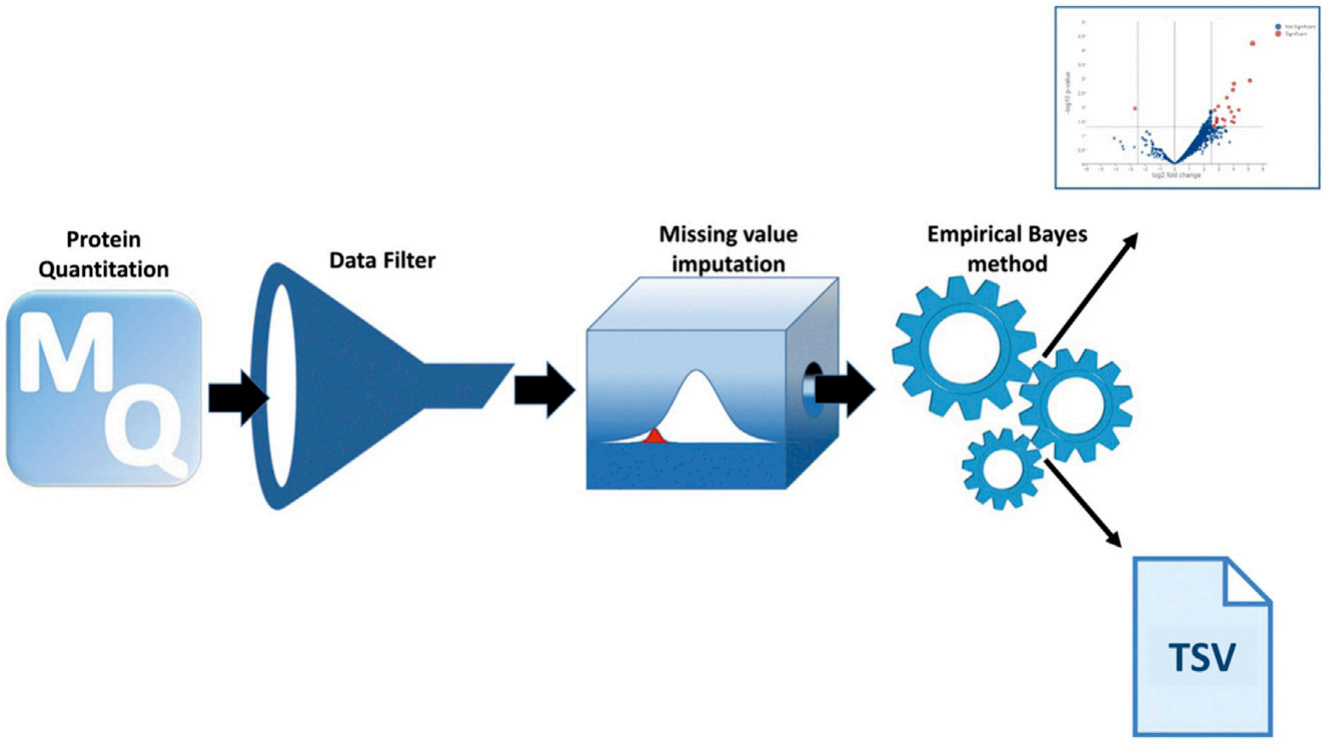
Statistical data analysis pipeline This pipeline implements statistical analysis of proteomic data in proteingroups.txt file obtained from MaxQuant search. First, data are cleaned by removing non-mitochondrial proteins. Then, after log transformation, the missing values are imputed. Finally, after employing an empirical Bayes method, moderated t statistics are computed, resulting in shrinkage of a protein’s variance toward a pooled estimate, thereby providing stable inference.
2. Make sure you have mitochondrial proteins (identified with UniProt ID) in your proteingroups.txt file otherwise the code will terminate with a message. For the list of experimentally determined mitochondrial proteins, See Mitochondrial_Proteins folder: https://github.com/wasimaftab/BioID_Proteomics/tree/master/Mitochondrial_Proteins.

***Note:*** The code can be modified for BioID analysis in other organisms, e.g., humans. To do so, replace the list of yeast mitochondrial proteins in https://github.com/wasimaftab/BioID_Proteomics/blob/master/Mitochondrial_Proteins/Experimentally_Determined_Mitochondrion_Proteins.txt with the most recent list of experimentally determined mitochondrial proteins for the organism (e.g., MitoCarta3.0 for humans (Rath et al., 2020)).

1. The pipeline depicted in Figure 3 is implemented in R programming language and the needed libraries are installed automatically.

3. First, we recommend to install RStudio v1.3.959 or higher and R v4.0.2 or higher in Windows OS. Then, run the *limma_main.R* code in RStudio by clicking **Source** in the latest version of RStudio and select a MaxQuant outputted *proteingroups.txt* file with either iBAQ/LFQ values (see *Example/MQ_output/proteinGroups_example.txt*). For our analysis we used iBAQ values. *limma_helper_functions.R*, the file containing the helper functions must be in the same directory as the *limma_main.R.*
4. There are two modes: use either full data or remove exclusive proteins before analysis. Proteins that have zero intensities in all the replicates of one group are defined as “exclusive proteins.” For example, a set of three “exclusively enriched” proteins in bait “Mrpl4” is defined in Table 1:

**Table 1:** Example of exclusively enriched proteins

iBAQ.Mrpl4_1	iBAQ.Mrpl4_2	iBAQ.Mrpl4_3	iBAQ.Kgd4_1	iBAQ.Kgd4_2	iBAQ.Kgd4_3	id	UniProt
114070	80945	134780	0	0	0	99	P40156
2198400	998140	760490	0	0	0	107	Q02888
91675	94831	106500	0	0	0	112	P46943

Proteins that do not participate in the two groups comparison are saved in the results folder as tsv files with their iBAQ/LFQ intensities.

5. The code is interactive, and it will help users to provide the correct names as input for bait and control as it appears in the proteingroups.txt file columns. Part of the name (case insensitive) is also accepted. It will then ask users if they want to median normalize their data prior to two-group comparison. if the median normalization is chosen, the code will boxplot (in RStudio “Plots” panel) the data before and after normalization. We did not choose to normalize as our replicates were highly consistent (see Figure 4). However, in case the replicate medians are not consistent it is recommended to median normalize them before performing two-group comparison. The code will also plot the data distribution (histogram) before and after missing value imputation.

**Figure 4:**
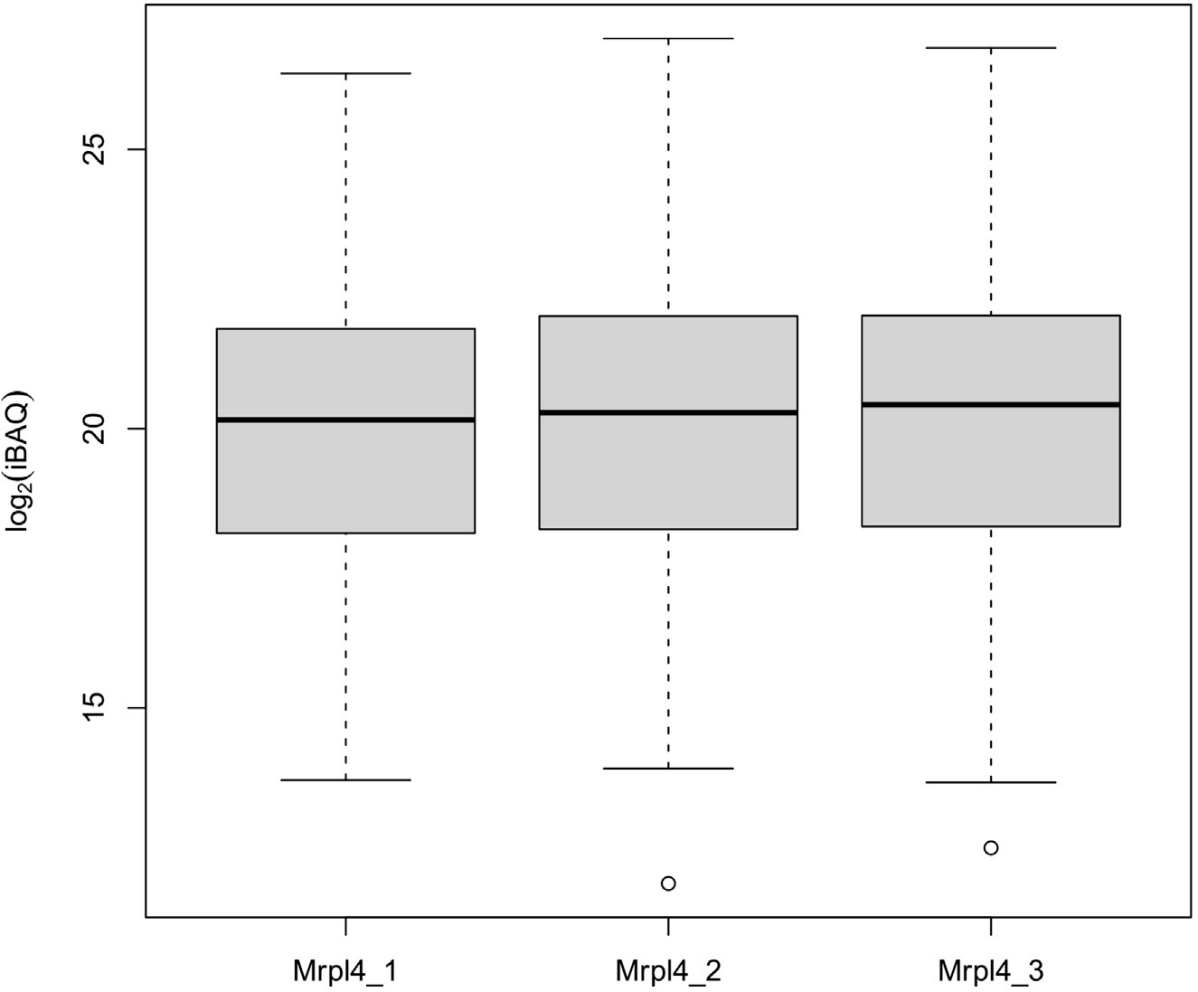
Mrpl4 replicates before median normalization Medians (using iBAQ values) of the three Mrpl4 replicates after data filtering and log transformation are 20.15, 20.28, 20.42, respectively, indicating the high data consistency and low inter replicate variability.
6. After a successful run, the code will create a volcano plot in html format and a tsv file containing final data inside a folder called "Results_timestamp" with the current system "timestamp" in the same directory where the limma_main.R file is present. Two such sample result folders can be found in *Example/Limma_output* folder: https://github.com/wasimaftab/BioID_Proteomics/tree/master/Example/Limma_output.
7. The plot can be visualized in any browser and saved as png file.
8. In addition, the code will save “exclusively enriched” proteins (if any) in control and bait replicates with corresponding LFQ/iBAQ values in the "Results_timestamp" folder.

The code was tested on 64 bit Windows 10 Enterprise (1909), Rstudio v1.3.959, R v4.0.2, and using the packages shown in Table 2.

***Note:*** Since the missing values are imputed randomly (from normal distribution), users can see minute change in numbers associated with fold change and p values across multiple runs of the pipeline. However, the major patterns in the data do not change.

***Note:*** The statistical data analysis pipeline relies on the user input for bait and control names, which should match the corresponding column names in the proteingroups.txt file. Therefore, name the samples carefully before submitting to mass spec to get a smoother data analysis. As an example, we named the three replicates of Mrpl4 as “Mrpl4_1”, “Mrpl4_2,” “Mrpl4_3”. Then, while running “*limma-main.R*,” we entered Mrpl4 as bait name and the R code was able to acquire data for all the 3 replicates of Mrpl4 without any ambiguity.

**Table 2:** List of packages required (installed automatically)

Package	Version
dplyr	≥0.8.3
stringr	≥0.4.0
MASS	≥7.3–5.4
plotly	≥4.9.0
htmlwidgets	≥0.3
limma	≥3.42.0
qvalue	≥2.8.0

## Limitations

BioID is a powerful tool to identify proteins localized in proximity of a bait. However, the size of the BirA∗ tag (∼38 kDa) might compromise the localization and the function of the bait protein. For example, the import in mitochondria could be affected. Hence, it is of high importance to verify the subcellular localization and the functionality of the tagged bait. Moreover, BirA∗ biotinylates primary amines. As consequence, if a nearby protein has no exposed primary amines, it will not be biotinylated and hence will not be detected by mass spectrometry.

## Troubleshooting

### Problem 1

Tagging BirA∗ to a bait protein may disrupt its molecular functioning. By utilizing non-functional bait proteins, we may identify false positive proximity interactions and/or miss genuine interactors. Hence, the resultant network would not reflect the true physiological context and the data may be considered unreliable and misleading.

### Potential solution 1

It is important to test the molecular functioning of the BirA∗-tagged bait by a robust and quick assay. In the case of Mrpl4 and Cbp3, a loss of their function would result in the loss of cellular respiration. Hence, we assayed for respiratory growth of the cell lines under non-fermentable carbon source, i.e., glycerol, and proceeded ahead with only those cell lines that displayed no growth defects.

### Problem 2

While running the statistical data analysis pipeline described in "Quantification and statistical analysis", user might encounter the following error: Error in plot.new() : figure margins too large

This happens when the plot pane has very little/no area to plot. Once you hit this error, code does not proceed further and gets killed.

### Potential solution 2

To solve this problem, user needs to increase the area in RStudio plot pane. The issue and its potential solution are presented in a video in the following link:

https://github.com/wasimaftab/BioID_Proteomics/tree/master/Troubleshooting/plot_issue.webm

## Resource availability

### Lead contact

Further information and requests for resources and reagents should be directed to and will be fulfilled by the Lead Contact Martin Ott (martin.ott@gu.se).

### Materials availability

Yeast strains generated in this study are available by contacting Martin Ott. Plasmids used in the study are available on Addgene, ID: 160290 and 160291.

### Data and code availability

The code required to perform statistical analysis on the BioID LC-MS data and to automate creation of Bait-Prey network from the analyzed datasets is available on GitHub repository: https://github.com/wasimaftab/BioID_Proteomics.

Mendeley dataset for western blots: https://doi.org/10.17632/pw4f8w65pk.1.
